# A Randomized Placebo-controlled Trial of Clonidine Impact on Sedation of Mechanically Ventilated ICU Patients

**Published:** 2015

**Authors:** Maryam Farasatinasab, Mehran Kouchek, Mohammad Sistanizad, Reza Goharani, Mirmohammad Miri, Mehrdad Solouki, Padideh Ghaeli, Majid Mokhtari

**Affiliations:** a *Department of Clinical Pharmacy, Faculty of Pharmacy, Shahid Beheshti University of Medical Sciences, Tehran, Iran. *; b *Department of Anesthesiology, Pain and Critical Care Medicine. Department of Anesthesiology, Shahid Beheshti University of Medical Sciences, Tehran, Iran. *; c *Emam Hossein Medical and Educational Center, Shahid Beheshti University of Medical Sciences, Tehran, Iran.*; d *Department of Medicine, Pulmonary and Critical Care Medicine, Shahid Beheshti University of Medical Sciences, Tehran, Iran.*; e *Department of Clinical Pharmacy, Faculty of Pharmacy, Tehran University of Medical Sciences, Tehran, Iran.*

**Keywords:** ICU Sedation, Clonidine, Morphine, Midazolam, Propofol

## Abstract

Clonidine has sedative and analgesic properties. Randomized studies examining these properties in mechanically ventilated ICU patients are scarce. This study was designed to assess the impact of clonidine on sedative agent use in mechanically ventilated patients.

In a prospective, randomized, double blind, placebo-controlled study in a general ICU of a university medical center in Tehran, Iran, 40 patients, over 18 years on mechanical ventilation for 3 days or more randomized into 2 equal groups of clonidine and placebo. Clonidine arm received usual sedation and enteral clonidine 0.1 mg TID and escalated to 0.2 mg TID on the second day if hemodynamics remained stable. Ramsay Sedation Score was used to assess sedation.

Opioids and midazolam were used in all patients. 10 patients in clonidine and 3 in placebo arms had history of drug abuse (P = 0.018). The mean of sedatives used in the clonidine/placebo arms (mg/day) were; MED (Morphine Equivalent Dose) 91.4 ± 97.9/112.1 ± 98.8 P=0.39, midazolam 7.1 ± 7.9/8.3 ± 9.2 P=0.66 and propofol 535.8 ± 866.7/139.1 ± 359.9 P=0.125. After adjusting for addiction and propofol, clonidine reduced MED use by 79.6 mg/day (P=0.005) and midazolam by 5.41 mg/day (P = 0.05).

Opioids and midazolam need reduced by clonidine co-administration regardless of history of drug abuse. Acceptable side effect profile and the lower cost of clonidine could make it an attractive adjunct to sedative agents in ICU.

## Introduction

Critically ill ICU patients, particularly those on mechanical ventilation are exposed to anxiety, pain and stress (1). Sedation, analgesia and delirium and their management are important aspects of care delivery in ICU with greater attention in recent years to research in sedation strategies, short term complications and longer term sequels (2, 3).

Commonly used parenteral sedative agents in ICU are benzodiazepines such as midazolam, lorazepam and diazepam; opioids such as morphine, fentanyl, remifentanil, sufentanil and alfentanil; propofol and dexmedetomidine. However each of these drugshas their own problems with possible added adverse effects when they have to be used in combination (4).

Benzodiazepines can be associated with respiratory depression and hypotension, especially if they are given in combination with other cardiopulmonary depressants. In addition, tolerance to benzodiazepines can develop in long-term administration. Propofol can cause dose-dependent respiratory depression, hypotension or in occasions propofol infusion syndrome (PRIS).

Dexmedetomidine, a newer α2 receptor agonist that has been approved for short-term (24 h) ICU sedation, is one of the most favored agents. Patients sedated with dexmedetomidine respond to stimuli and arouse easily with minimal respiratory depression (4). However this agent could be quite costly and may not be readily available particularly in third world countries, moreover ICU patients may require much longer periods of sedation than the approved 24 hours for dexmedetomidine (4).

Opioids are not currently favored as ICU sedative agents of choice (4), however they are widely used for their sedoanalgesic effects. Development of tolerance to these drugs could pose problems with their administration in ICU.

Clonidine, an older α2 receptor agonist is used to treat various conditions such as hypertension, menopausal flushing and withdrawal from opioids or alcohol. In addition clonidine has analgesic and sedative properties. Sedative effects of clonidine appear to be due to its action on locus ceruleus in brain stem.

Considering clonidine’s sedative properties and its safety profile studies exploring the use of this agent in ICU settings seems to be appropriate. To our knowledge there are no randomized-control studies examining sedative properties of clonidine in critically ill ICU patients.

We undertook this single centerrandomized-control trial to assess the impact of adding clonidine to commonly administered sedating agents in mechanically ventilated patients in a general intensive care unit of a teaching hospital.

## Experimental

*Patients and settings*

This study was carried out in the general ICU of a 550 bed university hospital in Tehran, Iran. A large variety of medical, surgical, orthopedics, neurosurgical, trauma, cardiac surgery and obstetrics and gynecology patients are managed in this hospital. The ICU care is directed and run by trained intensivists in a semi-closed fashion with average patientlength of stay of 8 days.

This prospective, randomized, double-blind, placebo-controlled study included 40 ICU patients over the age of 18 who required mechanical ventilation for three days or longer with stable hemodynamics and mental status during the study period.

Patients were excluded if they had sepsis based on surviving sepsis guideline (6), volume depletion, second or third degree atrioventricular node block, systolic blood pressure less than 90 mmHg, acute or chronic renal insufficiency (GFR <15 mL/min), severe liver failure based on Child-Pugh scoring system, GCS<8, received clonidine for less than 3 days after randomization, history of clonidine use during the past 90 days and inability to receive drugs enterally.

Baseline data recorded were age, sex, reason for ICU admission, APACHI II scores (7) and history of drug abuse. Laboratory data and daily events including systolic and diastolic blood pressure and heart rate were documented.

The depth of sedation was assessed by Ramsey sedation score (5) and optimal sedation was considered to have reached when patients were maintained between scores of 3 and 4.

*Study protocol*

Patients were randomized into two groups using simple randomization method. Our ICU sedative regimen for mechanically ventilated patients typically consists of fentanyl, midazolam, morphine and propofol.

The intervention group received the usual sedative agents prescribed by intensivists plus enteral clonidine 0.1 mg every 8 hours via NGT. The dose was escalated to 0.2 mg every 8 hours on the second day if hemodynamics remained stable. The control group received placebo in addition to the prescribed sedative regimen. This was continued for at least three days and in patients requiring longer duration of sedation the protocol was extended up to seven days.

We recorded the daily doses of sedative agents and Ramsay Sedation Scores for each patient. The doses of fentanyl and methadone were converted to morphine equivalent dose (MED) on the assumption that 10 µg of fentanyl and 1 mg of parenteral and 2 mg of enteral methadone was considered equivalent to 1 mg of morphine (8). Doses of lorazepam and alprazolam were converted to midazolam equivalents on the assumption that 1 mg of lorazepam and 0.5 mg of alprazolam were equivalent to 1.4 mg of midazolam (9). Finally the impact of clonidine on the total amount of sedative agents used and the quality of sedation were evaluated.

*Statistical analysis*

Statistical analysis was performed using SPSS® 19 Software. Continuous variables in each group of subjects were expressed as mean values ± standard deviation (SD). Differences between mean of two groups were performed using Mann-Whitney u-test. Chi-square test to compare dichotomous variable were used. Linear regression model was applied for assessment of addiction and propofol effect on opioid and midazolam consumption. In all cases a p-value of less than 0.05 was considered to be statistically signiﬁcant.

## Results

*Patient population*

Fifty five patients, 30 in the intervention and 25 in control groups were enrolled in the study. Ten patients were excluded in the treatment arm; five due to extubation under 3 days, two for administration errors, two due to hypotension and one who died on the second day of enrolment.

**Figure 1:**
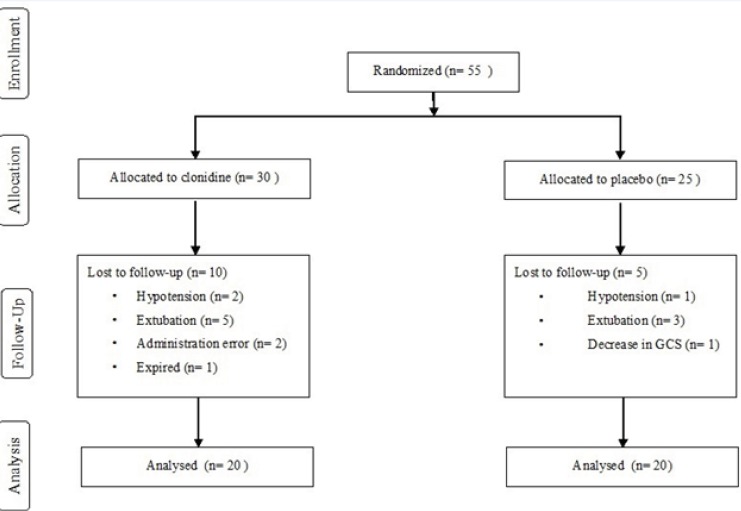
Flow chart of the study

Five patients were withdrawn from the study in the control arm; three due to extubation under 3 days, one with decreased level of consciousness and one for hypotension. The overall methodology for the study has been presented in the flowchart shown in Figure 1.

Basic patient characteristics, the severity of illness based on APACHI II scores measured in the first 24 hours of ICU admission and total duration of the study were not significantly different among two groups. Number of patients with drug abuse were 10 (50%) in intervention and 3 (15%) in the control group, P = 0.018 (Table 1).

**Table 1:** Demographic data of patients in intervention and placebo arms

		Total	Clonidine	Placebo	P
Age		57 ± 21	57 ± 25	58 ± 18	0.925‡
Sex	F	14 (35.0%)	5 (25.0%)	9 (45.0%)	0.185*
	M	26 (65.0%)	15 (75.0%)	11 (55.0%)	
Drug abuse	No	27 (67.5%)	10 (50.0%)	17 (85.0%)	0.018*
	Yes	13 (32.5%)	10 (50.0%)	3 (15.0%)	
APPACHI II Score		19 ± 5	19 ± 5	19 ± 4	0.818‡
Disease					
Multiple trauma CVA and neurologic problems Abdominal complication COPD DVT Insulin poisoning CHF Hysterectomy		15 9 7 5 1 1 1 1	7 5 2 3 1 1 1 0	8 4 5 2 0 0 0 1	
Duration of Study		5.9 ± 1.3	5.8 ± 1.4	6.1 ± 1.3	0.436‡
					

‡ Based on Mann-Whitney test.

* Based on Chi-square test.

*Clonidine effect on consumption of sedative agents*

Opioids and midazolam were administered to all patients in both groups. Propofol was used for eight and four patients in the treatment and control groups respectively.

The mean MED (mg/day) used in the clonidine arm was 91.4 ± 97.9 and placebo arm was 112.1 ± 98.8(P = 0.39) and after excluding 12 patients who received propofol it was 47.4 ± 67 and 101.7 ± 98.7 in the treatment and placebo arms respectively (P = 0.063).

**Figure 2:**
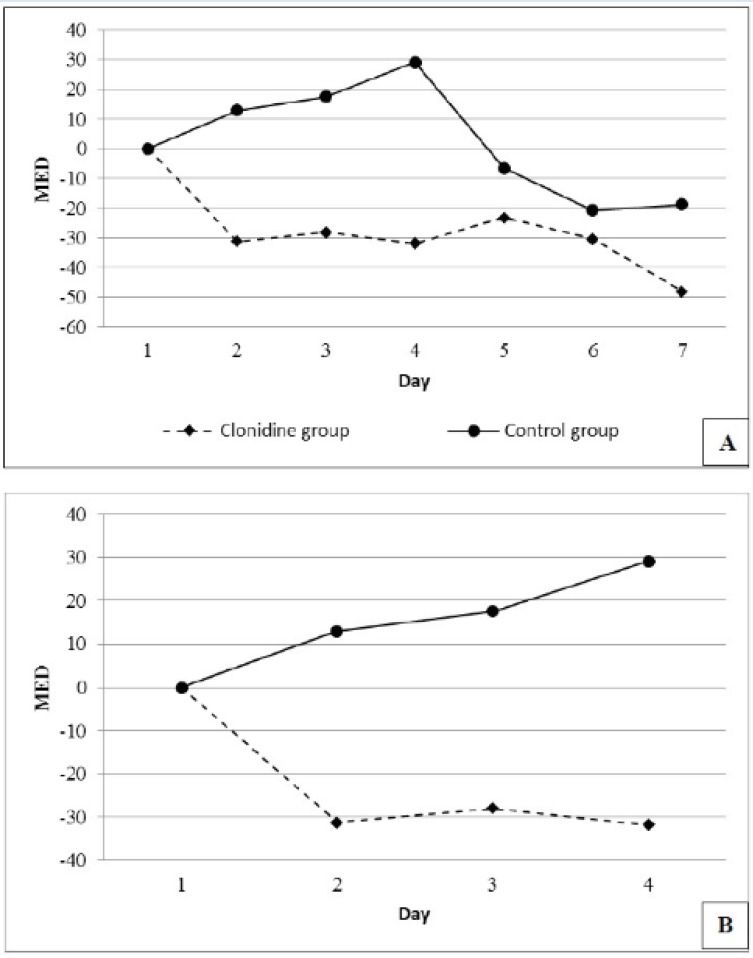
Mean daily change in morphine equivalent doses (MED) use for 7 days (panel A) and 4 days (panel B) of the study.

The number of patients requiring 4 days of MED were 19 (95%) and 18 (90%) and 7 days of MED were 13 (65%) and 11 (55%) in the placebo and the clonidine arms respectively. Mean daily MED use (mg/day) during the study period reduced by 27.5 ± 14.36 in the clonidine arm and increased by 1.96 ± 18.78 in the placebo arm (P=0.006) (Figure 2).

The mean doses (mg/day) of midazolam consumption were 7.1 ± 7.9 and 8.3 ± 9.2 in the intervention and placebo arms respectively (P = 0.66) (Table 2).

**Table 2:** Consumption of sedative agents with or without clonidine

Drug	**Clonidine**			**Placebo**			P‡
	Number of patients receiving clonidine	Mean ± SD	Median (Range)	Number of patients receiving placebo	Mean ± SD	Median (Range)	
Morphine*	20	91.4 ± 97.9	37 (8 to 295)	20	112.1 ± 98.8	96.5 (11 to 350)	0.394
Morphine without propofol	12	47.4 ± 67	21 (8 to 237)	16	101.7 ± 98.7	65 (11 to 350)	0.063
Propofol	8	535.8 ± 866.7	0 (0 to 2600)	4	139.1 ± 359.9	0 (0 to 1425)	0.125
Midazolam	20	7.1 ± 7.9	2.5 (0 to 20)	20	8.3 ± 9.2	5.5 (0 to 31)	0.668
Midazolam without propofol	12	3.5 ± 5.49	0.355 ( 0 to 16)	16	6.20 ± 7.45	3.98 (0 to 25)	0.30

‡ Based on Mann-Whitney test.

* All opioids equalized doses to morphine

**Figure 3:**
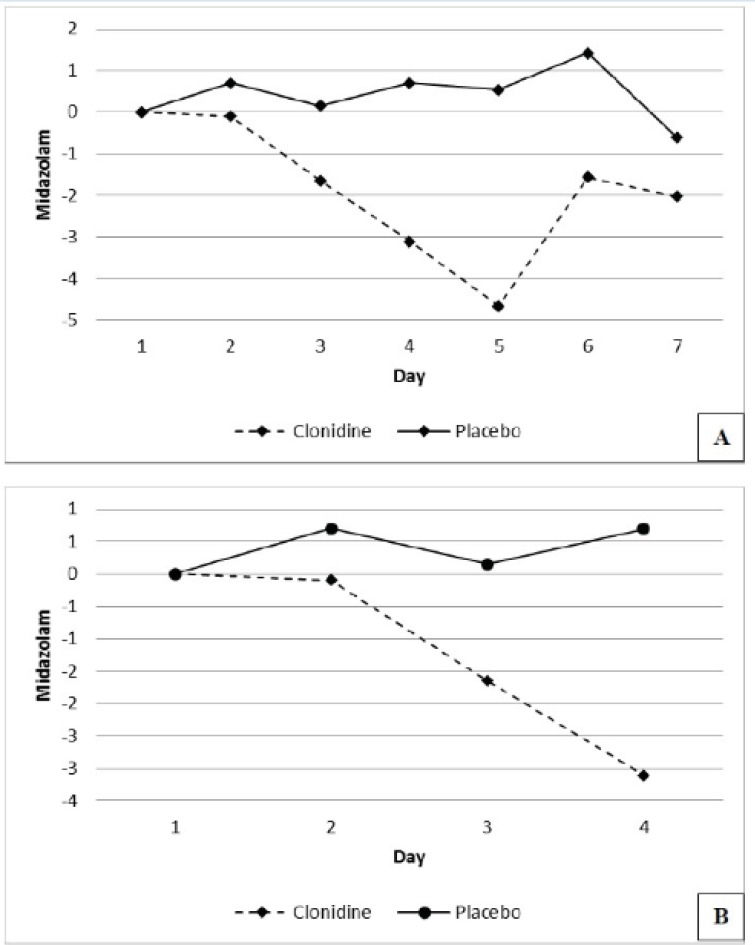
Mean daily change in midazolam use for 7 days (panel A) and 4 days (panel B) of the study.

The number of patients requiring 4 days of midazolam were 19 (95%) and 19 (90%) and 7 days of midazolam were 13 (65%) and 13 (65%) in the placebo and the clonidine arms respectively. The mean daily midazolam use (mg/day) during the study period reduced by 1.87 ± 1.63 in the clonidine arm and increased by 0.41 ± 0.64 in the placebo arm respectively (P=0.005), (Figure 3).

The mean doses (mg/day) of propofol used was 535.8 ± 866.7 in eight patients of intervention arm and 139.1 ± 359.9 in four patients in the control arm (P = 0.125).

*The effect of drug abuse on sedative requirement*

Ten patients in the treatment arm and three in placebo had history of drug abuse (P=0.018).

In patients without and with history of drug abuse the sedative requirements were; MED 64.1 ± 66.2 mg/day and 179.8 ± 108 mg/day (P=0.003), midazolam 5.6 ± 7.7 mg/day and 11.9 ± 8.7 mg/day (P=0.018) and propofol 276.7 ± 658.5 mg/day and 463.5 ± 748.7 mg/day (P=0.188).

Linear regression adjustment for the effects of confounding variables such as history of drug abuse and higher propofol use in the intervention arm, 8 versus 4, indicated a reduction in opioid requirement in the clonidine arm by 79.6 mg/day (P=0.005). After similar adjustments for the study groups and propofol use opioid requirement was noted to be 143.4 mg/day lower in patients with no history of drug abuse (P = <0.001).

Adjusting for clonidine and history of drug abuse did not indicate any significant effect of propofol on opioids use, P=0.24 (Table 3).

**Table 3:** Regression analysis for drug abuse effect and propofol on opioids requirement

**Group**		**Regression Coefficient**	**P**	**95% CI**	
				**Lower**	**Upper**
Clonidine	Yes	-79.6	0.005	-133.4	-25.8
	No	Reference	Reference	Reference	Reference
					
Propofol	Yes	0.02	0.24	-0.02	0.06
	No	Reference	Reference	Reference	Reference
Drug abuse	No	-143.4	<0.001	-198.8	-88.0
	Yes	Reference	Reference	Reference	Reference

Adjustment for propofol and drug abuse indicated a reduction of midazolam use by 5.41 mg/day in the presence of clonidine (P = 0.05). Similar adjustments for the study groups and propofol use showed a reduction of midazolam by 7.9 mg/day (P=0.008) in patients with no history of drug abuse. Adjusting for clonidine and history of drug abuse did not indicate any significant effect of propofol on midazolam use (P=0.07) (Table 4).

**Table 4:** Regression analysis for drug abuse effect and propofol on midazolam requirement

**Group**		**Regression Coefficient**	**P**	**95% CI**	
				**Lower**	**Upper**
Clonidine	Yes	-5.41	0.05	-10.96	0.13
	No	Reference	Reference	Reference	Reference
					
Propofol	Yes	0.003	0.07	0.000	0.007
	No	Reference	Reference	Reference	Reference
Drug abuse	No	-7.91	0.008	-13.62	-2.21
	Yes	Reference	Reference	Reference	Reference

*Clonidine adverse effect*

There were no significant differences between heart rate, systolic, diastolic and mean arterial blood pressure in the treatment and control group.

## Discussion

In our randomized double blind placebo-controlled study, we evaluated the effects of clonidine as a sedative adjunct in mechanically ventilated ICU patients. Clonidine was started in doses of 0.1 mg three times daily via feeding tube. This dose was escalated to 0.2 mg three times daily on the following day if hemodynamics remained stable.

The mean MED and midazolam requirement did not show any significant reduction in the clonidine arm in our initial analysis. However the analysis of daily changes in MED and midazolam use indicated a significant reduction of its requirement in the clonidine arm in subsequent days, particularly during the first four days of the study.

Adjustments for confounding variables such as history of drug abuse and unequal number of patients receiving propofol in the treatment and placebo arms, revealed a significant reduction of MED and a near significant reduction in midazolam requirement in the clonidine arm. (Tables 3 and 4).

The higher doses of midazolam used in day 6 were caused by an increased requirement for targeted sedation, by the 3 patients with history of drug abuse in the clonidine arm.

Opium, based on surveys in our patient population, is the commonest drug of abuse. We observed a significantly increased opioid requirement in patients with history of drug abuse in both arms of the study which is most likely secondary to the increased tolerance to opioids in these patients. 

Higher mean doses of propofol used in the treatment arm, albeit statistically insignificant, was due to the larger number of patients on tis agent than the placebo arm. This in turn, could have been due to the significantly higher number of patients with history of drug abuse, in the clonidine arm, with generally greater requirement for the targeted sedation.

Clonidine is a α2-adrenoceptor agonist introduced to the pharmaceutical market in 1960s for its antihypertensive effect (10). It has been used in varying doses and routes for other conditions such as migraine, menopausal flushing, drug and alcohol withdrawal, anesthetic adjuvant for sedation, analgesia, anxiety and autonomic dysfunction in sever tetanus (1, 4-11, 18-23).

Controlled studies examining the place of clonidine as an ICU sedative agent or adjunct to other agents are limited in medical literature.

The efficacy of clonidine for decreasing alcohol withdrawal symptoms in ICU patients on mechanical ventilation for over seven days was evaluated and published in an abstract form. In this study ten patients received clonidine intravenous infusion with fentanyl and midazolam infusion and 10 received fentanyl and midazolam infusion. Midazolam consumption was 34% lower in the treatment group. Sympathetic hyperactivity symptoms seen in 60% of placebo group were not observed in the clonidine arm. Forty multiple trauma patients on long-term mechanical ventilation with symptoms of sympathetic hyperactivity receiving fentanyl and midazolam for sedation, were studied by the same authors. Clonidine infusion led to decreased heart rate to less

than 120/min in 90% of patients within 24 hours with reduction of fentanyl and midazolam requirement by 38% ± 19% within 24 hours (15).

In another work, clonidine administration significantly decreased the withdrawal symptoms after sedation interruption in mechanically ventilated patients. This resulted in reduced respiratory, metabolic and hemodynamic demands and facilitated patient cooperation with the ventilator and weaning process (11).

In a small retrospective study of thirteen patients, clonidine was shown to reduce the opioid and benzodiazepine needs in ICU patients. In nine patients on opioids, the average daily dose was 44.7 mg morphine before and 28.2 mg after clonidine administration. In the eleven patients on lorazepam the daily dose was 14.6 mg pre and 3.9 mg post clonidine administration and six of seven patients receiving concomitant clonidine and propofol showed reduction in the requirement for propofol (16).

To our knowledge our study is the first randomized, placebo-controlled trial, examining the effects of predefined doses of clonidine co-administrated with other sedative agents in mechanically ventilated ICU patients.

Small sample size and simple randomization, which could not allow us better stratification of subgroups such as patients with history of drug abuse, are the main limitations of our study. Larger multicenter trials are needed to further elucidate our findings.

## Conclusions

Clonidine administration reduced opioids and benzodiazepines requirement significantly in mechanically ventilated ICU patients with or without history of drug abuse. However clonidine co-administration did not change propofol requirement. We did not observe any important adverse reactions of clonidine in the doses administered in our study.

Cost constraint and difficulties to access drugs such as dexmedetomidine, particularly in the third world countries and FDA current approval of this agent for only 24 hours could make clonidine a relatively safe and attractive adjunct to other ICU sedative agents.
